# Mutation spectrum of 122 hemophilia A families from Taiwanese population by LD-PCR, DHPLC, multiplex PCR and evaluating the clinical application of HRM

**DOI:** 10.1186/1471-2350-9-53

**Published:** 2008-06-20

**Authors:** Shin-Yu Lin, Yi-Ning Su, Chia-Cheng Hung, Woei Tsay, Shyh-Shin Chiou, Chieh-Ting Chang, Hong-Nerng Ho, Chien-Nan Lee

**Affiliations:** 1 Department of Medical Genetics, National Taiwan University Hospital, Taipei, Taiwan; 2 Institute of Biomedical Engineering, College of Medicine and College of Engineering, National Taiwan University, Taipei, Taiwan; 3 Department of Hematology, National Taiwan University Hospital, Taipei, Taiwan; 4 Department of Pediatrics, Kaohsiung Medical University Hospital, Kaohsiung, Taiwan; 5 Department of Biology, University of California, Los angels, USA; 6 Department of Obstetrics and Gynecology, National Taiwan University Hospital, Taipei, Taiwan

## Abstract

**Background:**

Hemophilia A represents the most common and severe inherited hemorrhagic disorder. It is caused by mutations in the F8 gene, which leads to a deficiency or dysfunctional factor VIII protein, an essential cofactor in the factor X activation complex.

**Methods:**

We used long-distance polymerase chain reaction and denaturing high performance liquid chromatography for mutation scanning of the F8 gene. We designed the competitive multiplex PCR to identify the carrier with exonal deletions. In order to facilitate throughput and minimize the cost of mutation scanning, we also evaluated a new mutation scanning technique, high resolution melting analysis (HRM), as an alternative screening method.

**Results:**

We presented the results of detailed screening of 122 Taiwanese families with hemophilia A and reported twenty-nine novel mutations. There was one family identified with whole exons deletion, and the carriers were successfully recognized by multiplex PCR. By HRM, the different melting curve patterns were easily identified in 25 out of 28 cases (89%) and 15 out of 15 (100%) carriers. The sensitivity was 93 % (40/43). The overall mutation detection rate of hemophilia A was 100% in this study.

**Conclusion:**

We proposed a diagnostic strategy for hemophilia A genetic diagnosis. We consider HRM as a powerful screening tool that would provide us with a more cost-effective protocol for hemophilia A mutation identification.

## Background

Hemophilia represents the most common and severe inherited hemorrhagic disorder. Hemophilia A(HA) is caused by mutations in the F8 gene, leading to a deficiency or dysfunctional III protein, an essential cofactor in the factor X activation complex. The F8 gene is 186 kb long; it has 26 exons and encodes a 9-kb mRNA transcript [1,2]. The mutations causing hemophilia A are spread throughout the gene and are mostly represented by point alterations. However, the inversion of intron 22 was found in 40–50% of patients with severe HA [3] and the inversion of intron 1 was reported with a prevalence of about 5% in the UK [4].

Patients suffering from the disorder, along with their families, bear great financial and social burden; therefore, it is very important to prevent recurrence of the disease. For the sake of genetic counselling and prenatal diagnosis of hemophilia A, it is necessary to establish a sensitive, rapid and economic genetic diagnostic system. However, comprehensive analysis of mutations in the F8 gene is difficult to conduct due to the large gene size, its many scattered exons, and the high frequency of de novo mutation. The most direct strategy for mutation detection would be to amplify these regions from genomic DNA using PCR [5]. However, it would necessitate almost 30 amplifications of genomic DNA to cover all the essential regions.

The first systematic analysis of the complete coding sequence of the F8 gene was performed by applying denaturing gradient gel electrophoresis (DGGE) after PCR amplification in 1991. The analysis demonstrated a 90% mutation detection rate [6]. Since then, a wide range of different mutations have been identified, providing the genetic basis for the extensive variability observed in the clinical phenotypes. Mutation detection in the F8 gene is so challenging that it is only partially met by conventional screening methods such as single stranded conformational polymorphism (SSCP), conformational sensitive gel electrophoresis (CSGE) and chemical mismatch cleavage (CMC), each with varying applicability and efficiency; however, they all suffer from incomplete detection rates in the range of 70–85% [7-11]. Moreover, each method places variable demands on the technical skills and time investment of the investigator. In contrast, the recently introduced denaturing high performance liquid chromatography (DHPLC) offers a promising new method for a fast and sensitive analysis(96.2%) of PCR-amplified DNA segment [12-15].

We have established a diagnostic strategy, consisting of screening for most common mutations in the F8 gene, using long-distance polymerase chain reaction (LD-PCR) and DHPLC. We reported the result of detailed screening of 122 Taiwan families with hemophilia A. In order to facilitate throughput and minimize the cost of mutation scanning, we also evaluated a new mutation scanning technique, high resolution melting analysis (HRM). This alternative screening technique detects sequence variation by the use of a saturating double-stranded DNA dye.

## Methods

### Patients

This study was approved by the Ethic Institute Review Board of National Taiwan University Hospital and included 122 families, in which 329 samples had hemophilia A family history. We obtained consent from each subject. The patients presented varying degrees of severity of the disease. The mild and moderate hemophilia A was diagnosed following the familial transmission analysis and after the elimination of von Willebrand disease type 2N either by FVIII:vWF binding test or sequencing of exon 18 to 24 of the von Willebrand gene.

### DNA extraction

According to the manufacturer's instructions, genomic DNA was extracted from 3 ml of peripheral blood cell samples with a Puregene DNA Isolation Kit (Gentra Systems, Minneapolis, MN).

DNA mutation numbering is based on cDNA sequence and nucleotide +1 corresponds to A of the ATG translation initiation codon. The nomenclature of this study follows the Nomenclature for Description of Genetic Variations approved by the Human Genome Variation Society and differs in 19 amino acids from the reference mutation database because the first 19 amino acids compose a signal peptide.

### Mutation identification

#### The PCR assay for intron 22 inversion

The PCR mixture contained a total volume of 50 μl: 1 mM 10× buffer, 76 mM DMSO, 0.5 mM of dNTP and 0.3 mM deaza-Dgtp(Amershan Biosciences; Freiburg Germany), 0.12 mM of Expand long Template DNA polymerase (Roches) and 0.36 mM each of primer P, Q, 0.06 mM each of primer A, B as described by Qiang Liu et al. [16,17]. Subcycling multiplex long distance PCR(LD-PCR) was carried out as described by Qiang Liu et al. [16,17] with slight modifications in the reaction temperatures. The conditions for LD-PCR for the first 10 cycles were set at 94°C for 12s, followed by four subcycles of annealing/elongation carried out at 62°C for 120s and 65°C for 120s. The remaining 20 cycles were modified by the addition of an extra 5s per cycle for each of the annealing/elongation step. 10 μl of PCR product were mixed with TAE buffer and electrophoresed for one hour on a 0.7% agarose gel, then stained with ethidium bromide and visualized under UV Light.

#### The PCR assay for intron 1 inversion

The PCR amplification of the F8 intron 1 region and the intron 1 h repeats were performed on 100 ng of genomic DNA, in a reaction volume of 25 μl containing 0.1 mM dNTP, 2 mM MgCl_2_, 10 mM buffer and 0.5 units Taq polymerase (Perkin Elmer, ABI Foster City, CA). The primers used for the amplification of the F8 intron 1 region were 0.12 mm of 9F, 9CR and int1h-2F each. Primers int1h-2F, int1h-2R and 9F were used for the amplification of intron 1 h repeats as previously described by Tizzano and Banagnall et al. [18,19]. After the initial denaturing step at 95°C for 5 min, thirty cycles of PCR were carried out designated at 94°C for 30s, 65°C for 30s and 72°C for 2 min. Finally, 5 μl of PCR products were mixed with TAE buffer and electrophoresed for one hour on a 0.7% agarose gel, then stained with ethidium bromide and visualized under UV light.

#### PCR assay and DHPLC for entire coding region

If there was no inversion in intron 1 or intron 22, we perform mutation analysis of the entire F8 coding region, including flanking splicing sites, by DHPLC using appropriate primers (Table 1). The DHPLC allows for the automated detection of single base DNA substitutions as well as small insertions and deletions. When using heteroduplex analysis by DHPLC under partially-denaturing conditions, heteroduplexes are retained less than their corresponding homoduplexes on a unique DNA separation matrix. DHPLC uses unpurified PCR products that are subjected to a final denaturing/reannealing step to ensure adequate formation of the heteroduplex. The cycling parameters of the amplification reactions were optimized for the fragment. Equal volumes of the polymerase chain reaction (PCR) product from the patient and from a wild-type control male were mixed, denatured at 95°C for 5 min and then incubated at 65°C for 30 min to obtain heteroduplexes. Mutational screening, performed on all amplified fragments from each patient, was carried out via denaturing high-performance liquid chromatography (DHPLC) on a Wave^® ^DNA Fragment Analysis System (Transgenomic Inc., San Jose, CA) with a DNASep column (Transgenomic). The DNASep column contains proprietary 2-mm nonporous alkylated poly (styrene divinylbenzene) particles. The DNA molecules eluted from the column are detected by scanning with a UV detector at 260 nm. DHPLC-grade acetonitrile (9017-03; J.T. Baker, Phillipsburg, NJ) and triethylammonium acetate (TEAA; Transgenomic, Crewe, UK) constituted the mobile phase [14,20]. For DHPLC analysis, heterozygous profiles were identified by visual inspection of the chromatograms.

**Table 1:** Primer pairs and PCR conditions for DHPLC analysis.

HA Primer pairs and PCR conditions for DHPLC analysis							
Exon primer	Sequence (5' to 3')	Length of PCR amplicon (bp)	Annealing Temp. (°C)	DHPLC oven (°C)		Elution profile(B%)	

1F	ACATCCAGTGGGTAAAGTTC	356	53	60	55	56–65	52–61
1R	AGACTTACATCCCCACAATC						
2F	TTGAAGTGTCCACCAAAATGAACGACT	211	54	59		48–57	
2R	GATACCCAATTTCATAAATAGCATTCA						
3F	GTACTATCCCCAAGTAACCTT	204	54	59		50–59	
3R	CATAGAATGACAGGACAATAGG						
4F	TACAGTGGATATAGAAAGGAC	296	54	57		54–63	
4R	TGCTTATTTCATCTCAATCCTACGCTT						
5F	CCTCCTAGTGACAATTTCCTA	188	54	55		49–58	
5R	AGCAGAGGATTTCTTTCAGGAATCCAA						
6F	CATGAGACACCATGCTTAGCT	224	54	60		51–60	
6R	AACTCTGGTGCTGAATTTGGAAGACCCT						
7F	CAGATTCTCTACTTCATAGCCATAG	324	54	58	56	55–64	53–62
7R	ATTAAAAGTAGGACTGGATA						
8F	ATATAGCAAGACACTCTGACA	338	54	58		55–64	
8R	AGAGAGTACCAATAGTCAAA						
9F	AGAGTTGGATTTGAGCCTACC	284	54	58	55	53–62	50–59
9R	CAGACTTTTTCTTCTTACCTGACCTT						
10F	GGATTTGATCTTAGATCTCGC	204	53	56	54	50–59	48–57
10R	ATTTTAGTTGTTATTGATGA						
11F	TTGAGCTATTTATGGTTTTG	294	53	58.5	56	53–62	51–60
11R	GACATACACTGAGAATGAA						
12F	GCATTTCTTTACCCCTTTCA	230	54	59.5	57	51–60	49–58
12R	CTTTATTCACCACCCACTG						
13F	TCCTGGGAATAAGATAATGG	393	54	57.5	56	56–65	54–63
13R	AGAGCATACGAATGGCTAGT						
14(I)F	ATCTGTGTTATGAGTAACCA	430	54	57.5		57–66	
14(I)R	TCATATTTGGCTTCTTGGAG						
14(II)F	CATGGGCTATCCTTATCTGA	479	54	56.5		57–66	
14(II)R	CATGAACTTTCTTGGCTATT						
14(III)F	TCAAAGTTGTTAGAATCAGG	441	54	55.5		56–65	
14(III)R	ATTTTGTGCATCTGGTGGAA						
14(IV)F	GTCCAACAGAAAAAAGAGGG	481	54	57	54.5	58–67	55–64
14(IV)R	CTACATTTTGCCTAGTGCTC						
14(V)F	CTGGCACTAAGAATTTCATG	429	54	57.5	56	57–66	55–64
14(V)R	CCTTCTCATTGTAGTCTATC						
14(VI)F	GAAACATTTGACCCCGAGCA	431	54	57.5		56–65	
14(VI)R	TTTTGGGCAAGTCTGGTTTC						
14(VII)F	CACATACAAGAAAGTTGAGA	436	54	59	57.5	56–65	55–64
14(VII)R	CTCATTTATTGCTGCTATTG						
14(VIII)F	GATACCATTTTGTCCCTGAA	415	54	57.5		56–65	
14(VIII)R	GTCACAAGAGCAGAGCAAAG						
15F	CACCTAGGAAAATGAGGATGT	300	53	58.5	56	53–62	51–60
15R	ATAGTCAGCAAGAAAATAAA						
16F	AAGATCCTAGAAGATTATTC	330	50	57.5		53–62	
16R	TTAGTACACAAAGACCATTT						
17F	TGATGAGAAATCCACTCTGG	349	54	58	56.5	54–63	53–62
17R	GTGCAATCTGCATTTCACAG						
18F	GTGGAATCCTCATAGATGTCA	312	53	57		54–63	
18R	GAGTAGGTAGAAGAAAGAGCAC						
19F	GCAAGCACTTTGCATTTGAG	305	52	59	56	55–64	52–61
19R	AGCAACCATTCCAGAAAGGA						
20F	CCATTTTCATTGACTTACATTTGAG	193	53	59.5	56.5	49–58	46–55
20R	AGATATAATCAGCCCAGGTTC						
21F	GAATTTAATCTCTGATTTCTCTAC	168	53	60.5	56	48–57	46–55
21R	GAGTGAATGTGAATACATTTCC						
22F	AAATAGGTTAAAATAAAGTGTTAT	206	53	48		48–57	
22R	GACTAATTACATACCATTAAG						
23F	CTCTGTATTCACTTTCCATG	250	54	57		52–61	
23R	ACAGTTAGTCACCCTACCCA						
24F	GCTCAGTATAACTGAGGCTG	249	54	58.5		51–60	
24R	CTCTGAGTCAGTTAAACAGT						
25F	AGTGCTGTGGTATGGTTAAG	323	56	58	56.5	55–64	54–63
25R	TTGCTCTGAAAATTTGGTCATA						
26F	ATCCTGGACTACTGGAAACA	393	53	63	58.5	56–65	51–60
26R	AGTTAATTCAGGAGGCTTCA						

Samples showing an abnormal peak pattern were sequenced using a fluorescent ABI Prism BigDye terminator kit (Applied Biosystems, UK), and the mutations were characterized. Each mutation was confirmed on a second, independent, amplified PCR sample. In order to rule out the possibility of exonic polymorphisms, 150 DNA samples from healthy, unrelated controls were screened and found negative for the novel missense mutations. All mutations found in the patients' family members were confirmed by sequencing.

### High Resolution Melting Analysis

Melting curve analysis with the high-resolution melting instrument is a simple, high-performance, reliable, high-resolution, time-saving, and low labor-intensive technique that has shown promise as a sensitive and specific tool for the detection of variations in DNA. The thermal stability of a PCR product is determined by its base sequence [21,22]. When the PCR product sequence is altered, duplex stability is changed, leading to different melting behavior. During high resolution melting analysis, melting curves are produced using dyes that fluoresce in the presence of double-stranded DNA and specialized instruments designed to monitor fluorescence during heating; as the temperature increases, the fluorescence decreases, producing a characteristic melting profile. The melting curve data were normalized, temperature-shifted, and converted to melting peaks by plotting the negative derivative of the fluorescence with respect to temperature against temperature (-dF/dT vs. temperature). This melting data can be analyzed to detect sequence variations such as single nucleotide polymorphisms and small insertions and deletions.

PCR amplification mixture included 2.5 mM MgCl_2_, 5 μl 2× Master Mix, 3.1 μl H2O (from Roche Reaction Mix Kit, Mannheim, Germany), 0.2 mM primers. And we added 25 ng DNA template. PCR amplification began with the first denaturation step at 95°C for 10 min, followed by 25 cycles of denaturation at 94°C for 30 s, annealing at 53°C for 45 s, extension at 72°C for 45 s, and then a final extension step at 72°C for 10 min.

Pipet 10 μl PCR products into each well of the LightCycler^® ^480 (Roche Applied Science) Multiwell Plate. Seal Multiwell Plate with LightCycler^® ^480 Sealing Foil. Load the multiwall Plate in LightCycler^® ^480 instrument and start the melting program. High resolution melting was performed at 95°C for 1 min, 40°C for 1 min, 6.5°C for 1 s and acquisitions at 95°C. The data was evaluated using the LightCycler^® ^480 Gene Scanning Software.

In order to improve the melting analysis, we redesigned the primers for some exons to shorten the PCR product and to avoid DNA-dimer formation (Table 2).

**Table 2:** Redesigned primer pairs for HRM

Exon	Oligo (5' – 3')	product size
HA – 1-1F	CCACTGATAAAAAGGAAGCA	226
HA – 1-1R	GTGGAGAGCTCTATTTGCAT	
HA – 1-2F	CCTCCTGGGAGCTAAAGATA	248
HA – 1-2R	CGATCAGACCCTACAGGA	
HA – 4-1F	CTTTGAGTGTACAGTGGATATAGAA	200
HA – 4-1R	AAAGATATGAGTAGGTAAGGCACA	
HA – 4-2F	TGATAAAGTCTTCCCTGGTG	209
HA – 4-2R	TGCTTATTTCATCTCAATCCT	
HA – 7-1F	CCTAGCAAGTGTTTTCCATT	246
HA – 7-1R	AGGTCCATCAAGAGTGTTTG	
HA – 7-2F	GCCACAGGAAATCAGTCTAT	236
HA – 7-2R	TTCATTTTAAAGATCCAAGA	
HA – 8-1F	TGAGCCAATTCAATCTCTTT	237
HA – 8-1R	ATCATCAAACCTGACCACAT	
HA – 8-2F	TAATGAAGAAGCGGAAGACT	245
HA – 8-2R	TTTTGAGTATGGGGAAGAGA	
HA – 9F	CCCAACCTCTCATCTTTTT	250
HA – 9R	CCAGACTTTTTCTTCTTACCTG	
HA – 11-1F	CAGATTTGTAGAACCCTTGC	216
HA – 11-1R	TAGAGTAATAGCGGGTCAGG	
HA – 11-2F	CTGCCAGGAGAAATATTCAA	239
HA – 11-2R	AAGGGGACATACACTGAGAA	
HA – 13-1F	CATGACAATCACAATCCAAA	244
HA – 13-1R	AGAATGGGAATAGGGTGAGT	
HA – 13-2F	GAGGTGGCATACTGGTACAT	219
HA – 13-2R	ATACGAATGGCTAGTGAAGC	
HA – 14-1F	CTGTGTTATGAGTAACCAGAGT	243
HA – 14-1R	CCTAGTGCTAGGGTGTCTTG	
HA – 14-2F	AATGCCATTGAACCAAGA	238
HA – 14-2R	CATATTTGGCTTCTTGGAGA	
HA – 14-3F	TCTTGCGACAGAGTCCTACT	242
HA – 14-3R	TTTCTTCAACTCTGTTGCTG	
HA – 14-4F	TCAGGCCTCCAATTAAGAT	250
HA – 14-4R	GGTCCACCAGACTCAGTAAG	
HA – 14-5F	AGA TAC CAC TCT ATT TGG CA	229
HA – 14-5R	AAC TTT GAA TAA GGC ATT A	
HA – 14-6F	TGGTAGGTTATTTAAAGGGAAA	248
HA – 14-6R	GCATTCTGTCATGAATCAAA	
HA – 14-7F	CCATCAGTCTGGCAAAATA	250
HA – 14-7R	ATCCACCTTGCTGATTCTG	
HA – 14-8F	CACAAAATCCAGATATGTCGT	235
HA – 14-8R	CCATCTCTTTGAGTCCTACG	
HA – 14-9F	AGGAAAGGGTGAATTTACAA	249
HA – 14-9R	TTTTGCCTAGTGCTCAGTAA	
HA – 14-10F	TTGCCTCAGATACATACAGTG	250
HA – 14-10R	TGCATGCATATTTCTCTACAA	
HA – 14-11F	AAAACTTGGAAGGCTTGG	250
HA – 14-11R	TTGTAGTCTATCTGTGTGAGG	
HA – 14-12F	GGTCCAAAAACATGAAACAT	245
HA – 14-12R	ATAAGATGCTGCTGGAAGAT	
HA – 14-13F	GGTCCTATTCCAAGACAACTC	250
HA – 14-13R	AGATGTTTTGGGCAAGTCT	
HA – 14-14F	CACATACAAGAAAGTTGAGAACA	250
HA – 14-14R	GCTTTCTGTTGCTACTCTCAG	
HA – 14-15F	TGGAATGAAGCAAACAGAC	250
HA – 14-15R	TTTGTCCCTCATTTATTGCT	
HA – 14-16F	ATACCATTTTGTCCCTGAAC	249
HA – 14-16R	TGTCAAAATCTTCCTTCTTCA	
HA – 14-17F	TTCAGTCAGATCAAGAGGAAA	246
HA – 14-17R	TCAAATGTCACAAGAGCAGA	
HA – 15F	TATTGCTTTTCCTCTGCTTT	214
HA – 15R	TTTCTTGTAATTCCACTGTCC	
HA – 16-1F	GGGATGTAAACCCTAAGGAC	228
HA – 16-1R	ATGATGTTGCACTTTCCAA	
HA – 16-2F	CTCTCGTCCCTATTCCTTCT	232
HA – 16-2R	TAAACCAAAAAGTGGTCAGC	
HA – 17-1F	ACTCATAGGATTGATGTCTTCC	238
HA – 17-1R	TAAAAGTGGGATCTTCCATC	
HA – 17-2F	CTAACACACTGAACCCTGCT	241
HA – 17-2R	TCATTTGTCAAAGTGCAATC	
HA – 18F	TTTTAACAGGCTTCTCTGTG	246
HA – 18R	AGGTAGAAGAAAGAGCACAAAC	
HA – 19F	AAATAATTTCTGTTCCTGTTG	222
HA – 19R	ATTCCAGAAAGGAAGAAAGC	
HA – 25-1F	GGT GAC CAA GAG GCT AC	170
HA – 25-1R	GAC TGC TGG AGA TGA GGA	
HA – 25-2F	CAG GGA GTA AAA TCT CTG C	155
HA – 25-2R	TGG TAT TTT TTT TCT TTC TT	
HA – 26-1F	AGAAGTGAGAAAAGCGTCTG	244
HA – 26-1R	ACCCTCAGTAGAGGTCCTG	
HA – 26-2F	GAATTCACCCCCAGAGTT	234
HA – 26-2R	AGAAATGCAGGACTGATGAT	

Some of the primers used for HRM were redesigned by LightCycler Probe Design Software 2.0. The primers not listed were the same with table 1

### Multiplex PCR by Capillary Electrophoresis

In one family, the band of exon 4 to exon 10 by electrophoresis after PCR was visible in female cases but not in affected male patients. Large deletion was suspected, so we designed multiplex PCR in order to differentiate normal female from female carriers.

Multiplex PCRs were used to optimize the system and allowed the amplification of exon 3 to exon11. The KRIT and FGFR2 genes were used as internal controls. Each multiplex PCR for the DNA fragments was performed in a total volume of 25 μL containing the following: 100 ng of genomic DNA; 0.1 to 0.5 μM each primer; 200 μM dNTPs; 1 unit of AmpliTaq Gold enzyme (PE Applied Biosystems); and 2.5 μL of GeneAmp 10× buffer II (10 mmol/L Tris-HCl, pH 8.3, 50 mmol/L KCl) in 2 mM MgCl_2 _as provided by the manufacturer. PCR amplification was carried out with an initial denaturation step at 95°C for 10 min, followed by 25 cycles of denaturation at 94°C for 30 s, annealing at 53°C for 45 s, extension at 72°C for 45 s, and then a final extension step at 72°C for 10 min.

The HDA system with GCK-5000 cartridge kit (eGene) was used to analyze exons of the F8 gene. The gel-matrix in the gel cartridge consists of proprietary linear polymer with ethidiumbromide (EtBr) dye. The PCR products were diluted 20-fold by deionized water and placed in the sample chamber of the instrument. The DNA samples were then automatically injected into the capillary channel and subjected to electrophoresis according to the manufacturer's operation protocol. BioCalculator Graphing software (eGene) enabled automatic labelling of the peak sizes.

## Results

Genomic DNA from 122 HA unrelated families were analyzed for mutations in the F8 gene (Table 3). There were 128 affected patients identified. This resulted in the identification of 61 different F8 gene mutations. The mutation detection rate was 100%.

**Table 3:** The detailed patient data.

Case number	Gender	Diagnosis	Result	Reported/Novel	HRM result	FVIII:C	Severity
69-1	M	Affected case	Exon 1 deletion	R		<1%	severe
61-1	M	Affected case	Exon 2, c.185, C>G, TCA>TGA, Ser>X	N		<1%	severe
109	M	Affected case	Exon 3, c.278, C>T, CCT>CTT, Pro>Leu	N	V	<1%	severe
82	M	Affected case	Exon 3, c.278, C>T, CCT>CTT, Pro>Leu	N		<1%	severe
144	M	Affected case	Exon 3, c.336_339, dup CCAT	N	indistinguishable	1.6%	moderate
71-1	F	Carrier	Intron 3, c.289-9 C>T	N	V		
42-3	M	Affected case	Exon 4, c.403 G>A, GAT>AAT, Asp>Asn	N		<1%	severe
59-1	M	Affected case	Exon 4, c.532, C>G, CTT>GTT, Leu>Val	N		<1%	severe
118-1	M	Affected case	Exon 4~Exon 10 deletion	R		1.1%	moderate
122	M	Affected case	Exon 7, c.829, A>G, ATT>GTT, Asp>Gly	N	V	12%	muld
114	M	Affected case	Exon 7, c.854_855, ins G	N	V	2.4%	moderate
79-1	M	Affected case	Exon 7, c.977 T>C, CTA>CCA, Leu>Pro	R		<1%	severe
94-1	M	Affected case	Exon 7, c.977 T>C, CTA>CCA, Leu>Pro	R		<1%	severe
16-3	M	Affected case	Exon 8, c.1247, C>T, CCC>CTC, Pro>Leu	N		4.2%	moderate
101	F	Carrier	Exon 9, c.1310 G>C, CGG>CCG, Arg>Pro	R	V		
72-1	M	Affected case	Exon 9, c.1315 G>A, GGT>AGT, Gly>Ser	R		3.0%	moderate
162	M	Affected	Exon 9, c.1336, C>T, CGA>TGA, Arg>X	R		4.8%	moderate
110	M	Affected case	Exon 9, c.1400, T>G, ATC>AGC, Ile>Ser	N	V	0.6%	severe
65	M	Affected case	Exon 11, c.1636, C>T, CGG>TGG, Arg>Trp	R		2.3%	moderate
92	M	Affected case	Exon 11, c.1648, C>T, CGC>TGC, Arg>Cys	R		12.0%	mild
136	M	Affected case	Exon 11, c.1678, A>G, AGA>GAA, Arg>Gly	N	V	11.0%	moderate
98	M	Affected case	Exon 12, c.1783_1790 del 8 mer	N	V	<1%	severe
119	M	Affected case	Exon 12, c.1804, C>T, CGA>TGA.Arg>X	R	V	1.5%	moderate
150-1	M	Affected case	Exon 12, c.1834, C>T, CGC>TGC, Arg>Cys	R		6.0%	mild
10-1	M	Affected case	Exon 14, c.2314 C>T, CAA>TAA, Gln>X	R		<1%	severe
104-1	M	Affected case	Exon 14, c.2605_2606, ins C	N	V	<1%	severe
113-1	M	Affected case	Exon 14, c.2609_2610, del CT	R	V	<1%	severe
85-1	M	Affected case	Exon 14, c.2939_2940, ins G	N	V	<1%	severe
124	M	Affected case	Exon 14, c.3294_3295, ins A	N	V	0.2%	severe
111	M	Affected case	Exon 14, c.3629, del A	R	V	<1%	severe
17-1	F	Carrier	Exon 14, c.3629, del A	R			
3-1	M	Affected case	Exon 14, c.3629, del A	R		<1%	severe
132	M	Affected case	Exon 14, c.4076 G>A, TGG>TAG, Trp>X	R		0.3%	severe
87-1	M	Affected case	Exon 14, c.4197 del C	N		3.0%	moderate
63-1	M	Affected case	Exon 14, c.4372, del A	N		3.6%	moderate
2-1	M	Affected case	Exon 14, c.4619, del T	N		<1%	severe
11-1	F	Affected case	Exon14, c.4814, C>A, TCA>TAA, Ser>X	R		2.0%	moderate
57-2	M	Affected case	Exon 14, c.4856, del C	R		<1%	severe
67-3	M	Affected case	Exon 14, c.4942 C>T, CAA>TAA, Gln>X			2.6%	moderate
115-1	M	Affected case	Exon 14, c.5069_5073, del 5 mer	R	V	<1%	severe
74	M	Affected case	Exon14, c.5143, C>T, CGA>TGA, Arg>X	N		<1%	severe
39-3	M	Affected case	Exon 15, c5343 T>A, TATA>TAA, Tyr>X	N		<1%	severe
48-5	M	Affected case	Exon 15, c.5353, G>A, GAA>AAA, Glu>Lys	N	V	3.7%	moderate
159-2	M	Affected case	Exon 16, c.5399, G>A, CGT>CAT, Arg>His	R		4.5%	moderate
97	M	Affected case	Exon 16, c.5465_5466, insA	N		4.9%	moderate
55-1	M	Affected case	Exon 16, c.5526, G>A, ATG>ATA, Met>Ile	R		2.1%	moderate
5-2	M	Affected case	Exon 16, c.5536, T>A, AAA>TAA, Lys>X	R		1.6%	moderate
123	M	Affected case	Exon 16, c.5576, A>G, GAT>GGT, Asp>Gly	N	indistinguishable	1.3%	moderate
161-1	F	Carrier	Exon 17, c.5711, A>G, GAG>GGG, Glu>Gly	N	V		
56-1	M	Affected case	Exon 17, c.5848, C>T, CGA>CAA, Gln>X	N		<1%	severe
1-1	M	Affected case	Exon 18, c.5879, G>A, CGA>CAA, Arg>Gln	R		7.4%	mild
133	M	Affected case	Exon 18, c.5879, G>A, CGA>CAA, Arg>Gln	R		5.5%	mild
107-1	M	Affected case	Exon 18, c.5953, C>T, CGA>TGA, Arg>X	R	V	0.8%	severe
138-2	M	Affected case	Exon 18, c.5953, C>T, CGA>TGA, Arg>X	R		<1%	severe
73	M	Affected case	Exon 22, c.6403, C>T, CGA>TGA, Arg>X	N		<1%	severe
14-2	M	Affected case	Exon 23, c.6506, G>A, CGT>CAT, Arg>His	R		25%	mild
20-1	M	Affected case	Exon 23, c.6506, G>A, CGT>CAT, Arg>His	R		2.9%	moderate
139-1	M	Affected case	Exon 23, c.6506, G>A, CGT>CAT, Arg>His	R	V	3.0%	moderate
125	M	Affected case	Exon 23, c.6532, C>T, CGC>TGC, Arg>Cys	R		6.5%	mild
170-1	M	Affected case	Exon 23, c.6532 C>T, CGC>TGC, Arg>Cys	R	V	8.9%	mild
174-1	M	Affected case	Exon 23, c.6532 C>T, CGC>TGC, Arg>Cys	R	V	8.4%	mild
95-1	M	Affected case	Exon 23, c.6545, G>A, CGC>CAC, Arg>His	R	V	2.5%	moderate
38-3	M	Affected case	Exon 24, c. 6575 G>T, AGT>ATT, Ser>Ile	R		7.2%	mild
102-1	M	Affected case	Exon 24, c.6671, C>T, CCT>CTT, Pro>Leu	R	V	2.7%	moderate
12-1	F	Affected case	Exon 24, c.6683 G>A, CGA> CAA, Arg>Gln	R		<1%	severe
41-3	M	Affected case	Exon 25, c.6724, G>A, GTG>ATG, Val>Met; Exon 8, c.1172, G>A, CGC>CAC, Arg>His	R;R	indistibuishable; V	<1%	severe
84-1	M	Affected case	Exon 25, c.6794_6795 del AG	R	indistinguishable	<1%	severe
93-1	M	Affected case	Intron 1 inversion	R		12.0%	mild
21-1	M	Affected case	Intron 22 inversion/Exon14 c.4531, G>A, GTT>ATT, Val>Ile	R;N	V	<1%	severe
100	M	Affected case	Intron 22 inversion	R		<1%	severe
103-1	F	Carrier	Intron 22 inversion	R			
105	M	Affected case	Intron 22 inversion	R		<1%	severe
106-1	M	Affected case	Intron 22 inversion	R		<1%	severe
112-1	M	Affected case	Intron 22 inversion	R		1.2%	moderate
116-1	M	Affected case	Intron 22 inversion	R		<1%	severe
117	M	Affected case	Intron 22 inversion	R		3.0%	moderate
120-1	M	Affected case	Intron 22 inversion	R		2.0%	moderate
121	M	Affected case	Intron 22 inversion	R		0.6%	severe
126-1	M	Affected case	Intron 22 inversion	R		<1%	severe
131-1	M	Affected case	Intron 22 inversion	R		<1%	severe
13-4	M	Affected case	Intron 22 inversion	R		<1%	severe
140	M	Affected case	Intron 22 inversion	R		1.1%	moderate
143-1	M	Affected case	Intron 22 inversion	R		3.0%	moderate
147	M	Affected case	Intron 22 inversion	R		0.5%	severe
149-2	M	Affected case	Intron 22 inversion	R		<1%	severe
151	M	Affected case	Intron 22 inversion	R		1.4%	moderate
15-1	M	Affected case	Intron 22 inversion	R		<1%	severe
152	M	Affected case	Intron 22 inversion	R		2.1%	moderate
153	M	Affected case	Intron 22 inversion	R		1.8%	moderate
154	M	Affected case	Intron 22 inversion	R		0.5%	severe
155	M	Affected case	Intron 22 inversion	R		1.7%	moderate
156	M	Affected case	Intron 22 inversion	R		1.8%	moderate
164	M	Affected case	Intron 22 inversion	R		<1%	severe
167	M	Affected case	Intron 22 inversion	R		<1%	severe
181	M	Affected case	Intron 22 inversion	R		3.0%	moderate
31-7	F	Carrier	Intron 22 inversion	R			
33-3	M	Affected case	Intron 22 inversion	R		<1%	severe
34-1	M	Affected case	Intron 22 inversion	R		<1%	severe
35-1	M	Affected case	Intron 22 inversion	R		3.0%	moderate
40-2	M	Affected case	Intron 22 inversion	R		<1%	severe
47-1	M	Affected case	Intron 22 inversion	R		3.2%	moderate
58-3	M	Affected case	Intron 22 inversion	R		<1%	severe
60-1	M	Affected case	Intron 22 inversion	R		1.6%	moderate
6-1	M	Affected case	Intron 22 inversion	R		<1%	severe
51-1	M	Affected case	Intron 22 inversion	R		2.7%	moderate
62-1	M	Affected case	Intron 22 inversion	R		<1%	severe
64-2	M	Affected case	Intron 22 inversion	R		<1%	severe
68-1	M	Affected case	Intron 22 inversion	R		<1%	severe
70-1	M	Affected case	Intron 22 inversion	R		<1%	severe
75-2	M	Affected case	Intron 22 inversion	R		<1%	severe
76-1	M	Affected case	Intron 22 inversion	R		<1%	severe
77-1	M	Affected case	Intron 22 inversion	R		<1%	severe
81	M	Affected case	Intron 22 inversion	R		<1%	severe
86-2	M	Affected case	Intron 22 inversion	R		<1%	severe
88-1	M	Affected case	Intron 22 inversion	R		<1%	severe
89-1	M	Affected case	Intron 22 inversion	R		<1%	severe
90	M	Affected case	Intron 22 inversion	R		0.8%	severe
91	M	Affected case	Intron 22 inversion	R		1.1%	moderate
9-1	M	Affected case	Intron 22 inversion	R		<1%	severe
96-2	M	Affected case	Intron 22 inversion	R		0.5%	severe
99	M	Affected case	Intron 22 inversion	R		<1%	severe
148	M	Affected case	Intron 22 inversion	R		<1%	severe

### Screening of the F8 intron inversion

**Figure 1:**
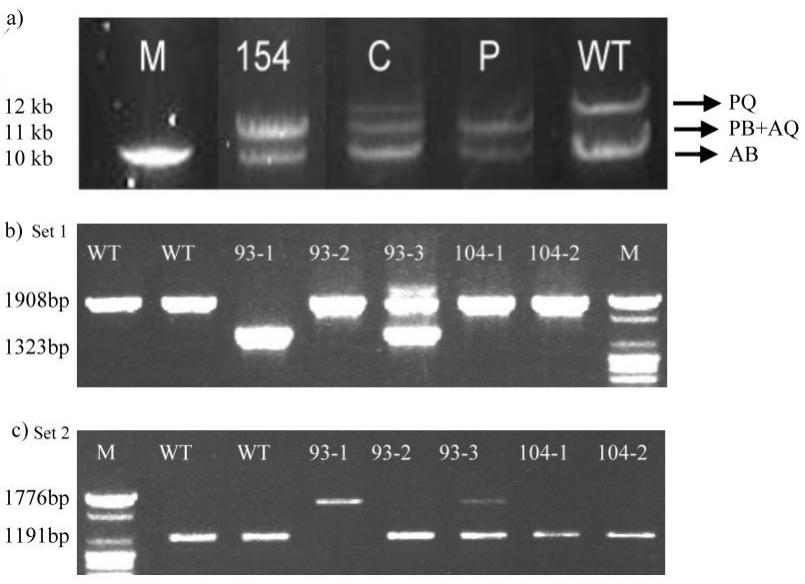
**Result of inversion detection of intron 1 and intron 22**. a) LD-PCR result of intron 22 inversion. Wild type has two band with PQ segment (12 KB) and AB segment (10 KB). With intron 22 rearrangement, PQ segment will be interrupted so that PB and AQ (both 11 KB) form. That is why we can see all three bands in carrier. Patient will show only 11 KB and 10 KB bands. Patient 154 is proved to be an affected case. M, marker. C, carrier. P, known affected male patient. WT, wild type. b) PCR result of intron 1 inversion. Set 1 is the product of primer 9F, int-2F and 9CR. In wild type, the only segment amplified is 1908 bp. In the affected male patient with intron 1 inversion, the amplified segment is 1323 bp in length. c) Set 2 is the product of primer 9F, int-2F and 2R. 93-2, 104-1 and 104-2 are normal. 93-1 is proved to be an affected case. 93-3 is a carrier with heterogenous components.

There were fifty-three (42.7%) families with intron 22 inversion (Fig. 1a) and one (0.8%) family with intron 1 inversion (Fig. 1b &1c) in this study. Since the intron 1 and the intron 22 inversion were well established causative mutations [3], there would be no need to search for other mutations in the coding regions in these families.

### Screening of the *F8 *mutation by DHPLC and direct sequencing

**Figure 2:**
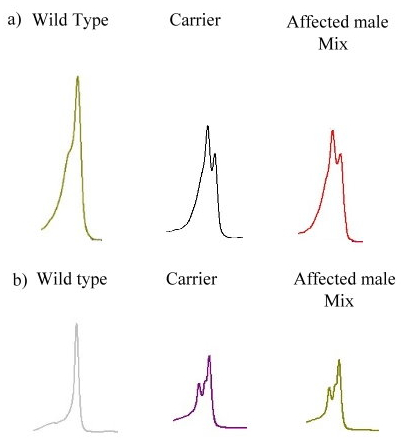
**Result of DHPLC**. DHPLC results of two families were illustrated. a) Family 85. b) Family 139. The wild type DNA showed homoduplex. The carrier DNA would show heteroduplex peak just as affected male after mixing with normal DNA.

We performed DHPLC for mutation screening in those families who didn't have intron 1 and intron 22 inversions. By DHPLC, 100% genetic mutation was detected in the 119 HA families and confirmed by sequencing. The two X chromosomes from the carriers are responsible for the heteroduplex formation of the PCR amplicons. These stutter peaks (heteroduplex) were eluted much earlier in the chromatographic process than the main peaks (homoduplex). Yet, the affected male patients with one X chromosome would not form a heteroduplex until it is mixed together with the wild type DNA (Fig. 2).

### Evaluating of High-Resolution Melting Analysis

In order to evaluate the HRM analysis, 25 families, including a total of 43 samples with known mutation and 341 normal controls were tested. The mutations consisted of 13 different missense mutations, 5 deletions, 4 insertions, 2 nonsense mutations, one splicing site nucleotide substitution and one duplication.

**Figure 3:**
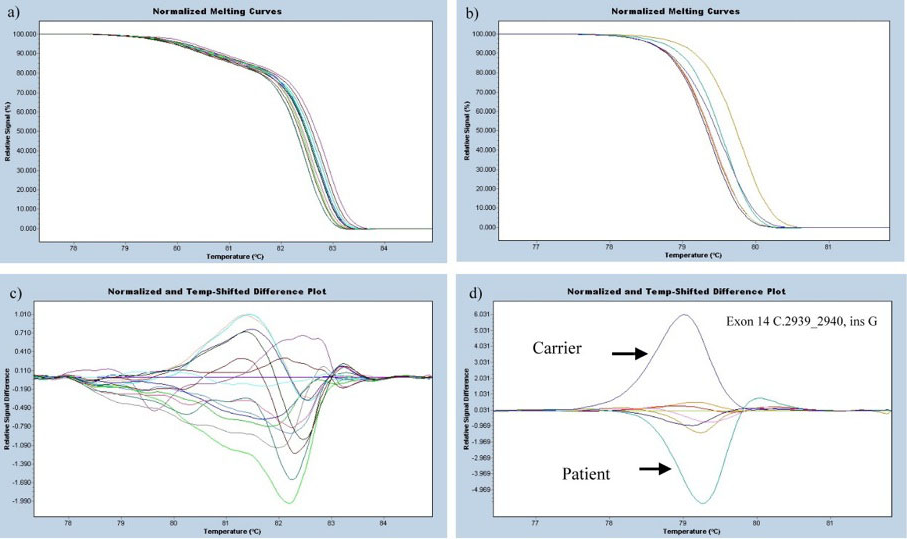
**Comparison of the results of different HRM primers**. a) and c) are HRM plots for exon 14-5 with first-designed primer. We are unable to see the grouping in the normalized, temperature-shifted plot. So we redesigned the primer pairs by LightCycler Probe Design Software 2.0. The result is shown in b) and d). The carrier and the patient are easy to be distinguished in the latter.

In order to avoid DNA-dimer formation, we redesigned the primers for the HRM PCR reaction. The resulting melting curves were easier to differentiate from normal controls and the sensitivity was elevated. Figure 3 compares the melting curves of the original and modified primers, respectively. As temperature increased, the double strand DNA was unwound and the fluorescence was released (Fig 3a and 3b). The melting curves were normalized by calculation of the "line of best fit" in between two normalization regions before and after the major fluorescence decrease representing the melting of the PCR product using the software the LightCycler^® ^Gene Scanning Software 4.0. The samples with high signal difference would present different peaks from the wild type (Fig 3d).

The different melting curve patterns were easily identified in 25 out of 28 cases (89%) and 15 out of 15 (100%) carriers. The sensitivity of HRM in this study was 93 % (40/43).

In addition, we performed HRM for whole coding region scanning in three families with unknown mutations. Three abnormal melting curves were identified. After these exons were confirmed by direct sequencing, we were able to identify the mutation in these three families. The specificity of HRM in this pilot study was high but it required further investigation. A larger sample sizes was needed for validation.

### Multiplex PCR

**Figure 4:**
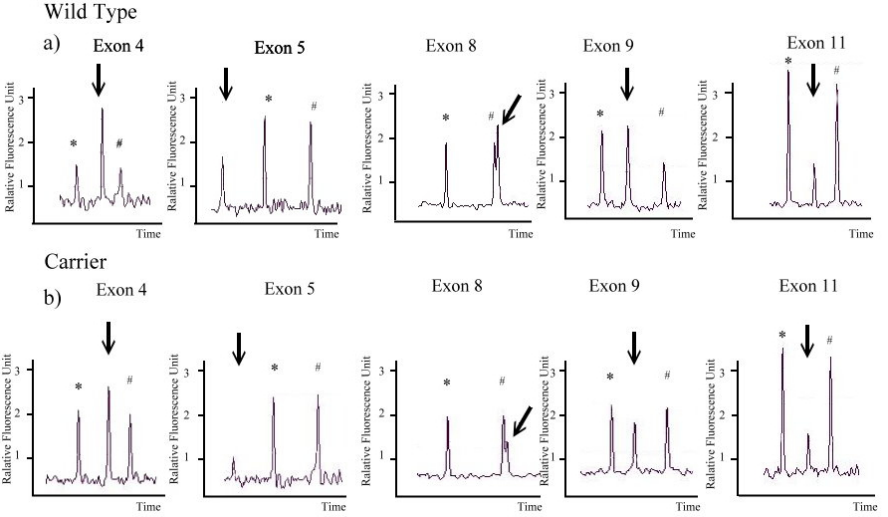
**Multiplex PCR in a family with exon 4 to exon 10 deletion**. FGFR2 (*) and KRIT (#) gene are used as internal control. a) are from wild type and b) are from female carrier. The X axis was time, which also represented the size of the amplicon. The Y axis was the fluorescence intensity of each amplicon, which also represented the relative gene dosage. The relative peak height of the female carrier with arrow is half to that of the wild type in exon 4, 5, 8,9 but not in exon 11.

There were two families, which we were unable to see the exon 4 to exon 10 band and the Exon1 band from electrophoresis after PCR, respectively. For the former, we designed the multiplex PCR that showed a total deletion from exon 4 to exon 10 (Fig 4). The ratio of the defective exon to the control gene in peak heights (the exon 4 to exon 10/the KRIT and the exon 4 to exon 10/the FGFR2) in the female carrier was half of that of the wild type. That is, the relative gene dosage in exon 4 to the exon 10 of the wild type was two times to that of the female carrier. As for the other family, we were unable to perform the multiplex PCR without having the DNA of the obligate carrier.

In this study we identified twenty-eight missense mutations, thirteen nonsense mutations, one four- base pair duplication, five insertions, nine small deletions, one splicing site nucleotide substitution and two large segment deletions in the F8 gene.

We identified twenty-nine novel mutations of the F8 gene in hemophilia A patients, in which eleven were missense mutations, one was splicing site substitution, seven were nonsense mutation, which lead to premature terminating of the protein, and ten were frameshift mutations including one four-base pair duplication, four deletions and five insertions, resulting in truncations of the F8 protein. None of the novel missense mutations were presented in the 150 normal DNA samples.

## Discussion

The most common mutation, found in approximately 40% of patients with severe hemophilia A, was the intron 22 inversion mutation [23]. This mutation happens when homologous recombination occurs between the Int22h-1 in intron 22 and 1 of 2 homologous regions (Int22h-2 or Int22h-3) telomeric to the F8 gene. An additional inversion of intron 1 of the factor VIII gene that affects up to 5% of patients with severe hemophilia A has been reported [18]. The large size of the F8 gene predisposes to the occurrence of deletions, which account for approximately 5% of characterized mutations. Single base pair changes (resulting in missense, frameshift, or splice junction mutations), insertions, or duplications account for the balance of cases of hemophilia A that are spread throughout the F8 gene [24]. Although the structure-function relationships of some of the missense mutations are known or can be deduced (eg, alteration of vWF binding site, thrombin cleavage site, etc), the structural consequences of most such mutations remain undefined [25].

Our data showed a mutation detection rate at 100% out of 122 hemophilia A families. Since the intron 22 inversion and intron 1 inversion present in 45.1 % in this Taiwanese database, 47.5 to 53% in India population [8,26] and 40–50% in European [3,18], it is rational to perform the inversion detection of intron 1 and intron 22 at first, and then genetic scan if no intron 1 or 22 inversion were found. In our cohort, 54.5% severe HA families had intron 1 and intron 22 inversion; 65.5% cases with intron 1 and intron 22 inversion belonged to severe hemophilia A patients. It is also suggested to check for intron 1, intron 22 inversion and big deletions in cases having the severe phenotype; on the other hand, it is recommended to go directly to the F8 gene mutation scanning in cases having the moderate/mild phenotype [25,27]. However, we are a referred center and the clinical data is not always available. Because the inversion detection of intron 1 and intron 22 is cheaper and more time-saving than other techniques, our strategy seemed to be economical under this situation.

The B domain accounts for 40% of the F8 cDNA. Apart from large exon or intron deletions, fourteen (14/52, 26.9%) different mutations and one SNP were found to be located in the B domain of the F8 cDNA. All mutations belonging to deletion, insertion, or nonsense mutations, lead to different protein products. This may imply the low functional importance of this region since no phenotype would be identified unless the protein product is changed or truncated. The SNP identified in the B domain was located in c. 3780 on exon 14, which changed aspartic acid to glutamic acid. Through family studies in a Caucasian population, Machiah et al. hypothesized that Asp1241Glu influences about 5% F8 levels and modifies thrombosis risk by modulating the rate of secretion into the circulation by family studies [28]. Scanavini et al. also conducted a case-control study and demonstrated that the F8 D1241E polymorphism was associated with decreased F8 activity [29].

Affected males with large deletions can be readily detected by the absence of an amplification product in PCR. Up to 98% of all frequent deletions can be detected, but in carriers, the nondeleted X chromosome hampers detection, making identification of hemophilia A carrier status difficult. In one particular family, the PCR product of an affected male patient failed to show exon 4 to exon 10. But of the same family, the same exon region in the female cases was successfully amplified by PCR and sequenced. Large deletion was suspected, but we were unable to distinguish the female carriers from the wild type. We designed the multiplex PCR [12,15] so that we could differentiate between them by examining different peak heights with the use of different gene dosages(Fig 4). In addition, the absence of peaks from exon 4 to exon 10 confirmed the deletion in the affected male. Multiplex PCR is an in-house designed reaction, which compares the dosage of several genes. This system may also be adapted for diagnostic use in other genetic diseases involving deletion and duplication mutations.

We had two heterozygous females from two unrelated families. Hemophilia A is transmitted through heterozygous females denoted as carriers, who are generally asymptomatic. This is because random X inactivation results in approximately equal proportions of somatic cells, in which either the normal X or the mutated X chromosome is active [23,30]. Because females have two X chromosomes and only 5% of FVIII is enough for the body to maintain hemostasis, it is very rare to have a female HA patient with very low FVIII activity and severe bleeding symptoms [31]. Even though the disease is extremely rare in females, a few cases have been documented as a result of different pathophysiologic mechanisms [32-34]. The skewed X-chromosome inactivation is the most common possible mechanism for the phenomenon [31,35-39].

The hemophilia A mutation screening is laborious and expensive, even when employing DHPLC as a screening method. In order to facilitate throughput and minimize the cost of hemophilia A mutation screening, we evaluated a new mutation scanning technique, high resolution melting system(HRM). We performed high resolution melting analysis on 43 samples, including carriers and affected cases. All the carriers were easily to be distinguished (100%). Nevertheless, we were unable to identify three out of 28 affected cases. The detection rate was 93%. We had a reason to believe that it is easier for the heteroduplex to release double-stranded DNA dye, so that HRM is more sensitive to carrier detection. DHPLC is not ideal in detecting affected case until mixed together with normal DNA. On the other hand, even though HRM was able to distinguish the affected male from the wild type, the sensitivity did not reach 100% despite optimizing condition. Therefore, mixing up the affected male DNA with normal control would improve the detection rate for both DHPLC and HRM. Besides, we proved that the analysis was greatly enhanced by good primer design that avoided DNA-dimer formation. The sensitivity of the analysis was not only influenced by the size of PCR products but also the primers and the PCR condition. We recommend the software, LightCycler Probe Design Software 2.0, as the tool for primer design.

As for clinical application, once the intron 1 and 22 inversion has been ruled out, HRM becomes a good initial screening method, especially for those families with obligate carriers. Comparing with our familiar method, DHPLC, HRM costs less ($0.6 versus $3.0 per sample per amplicon, respectively), takes less time (3 hours versus 10 hours) and works more efficiently (96 or 384 wells at one time). The analysis of HRM takes only fifteen minutes. With this highly sensitive and specific tool, we will be able to manage a large quantity of clinical samples and do mutation screening.

## Conclusion

In conclusion, we are capable of detecting 100% of mutations in the F8 gene; also our study identified a number of novel mutations in the largest cohort of Taiwanese patients yet to be reported. Furthermore, we validate HRM for hemophilia A mutation screening. Mutation detection is best started with obligate carriers. We proposed a cost-effective screening protocol with sequential combination of different genetic diagnostic tools. HRM saves time and money, and we have proven the use of such technique to be effective for mutation screening.

## Abbreviations

DHPLC: Denaturing High Performance Liquid Chromotography; F8 gene: FVIII gene; HA: Hemophilia A; HRM: High resolution Melting; LD-PCR: Long-distance PCR

## Competing interests

The authors declare that they have no competing interests.

## Authors' contributions

S–YL and C–CH carried out the molecular genetic studies. WT and S–SC participated in the clinical diagnosis, sequence alignment and sample collecting. S–YL and C–TC drafted the manuscript. C–CH, H–NH and Y–NS participated in the design of the study and performed the statistical analysis. C–NL conceived of the study, and participated in its design and coordination and helped to draft the manuscript. All authors read and approved the final manuscript.

## Pre-publication history

The pre-publication history for this paper can be accessed here:


